# Determination of Fluoride in the Bottled Drinking Waters in Iran

**Published:** 2010

**Authors:** Massoud Amanlou, Maedeh Hosseinpour, Homa Azizian, Mohammad Reza Khoshayand, Mojtaba Navabpoor, Effat Souri

**Affiliations:** a Department of Medicinal Chemistry, Faculty of Pharmacy and Pharmaceutical Sciences Research Center, Tehran University of Medical Sciences, Tehran, Iran.; b Department of Food and Drug Quality Control, Faculty of Pharmacy, Tehran University of Medical Sciences, Tehran, Iran.; c Faculty of Paramedical Sciences, Shahid Beheshti University of Medical Sciences, Tehran, Iran.

**Keywords:** Fluoride, Bottled drinking water, Ion-selective electrode, Fluoride determination, Dental carries

## Abstract

Fluoride is recognized as an effective agent for dental caries prevention. Generally, the main source of fluoride intake is drinking water. In this study, fluoride content in 18 commercial brands of bottled waters was investigated. Six samples from each batch of 18 Iranian commercial brands of bottled waters were supplied. The fluoride content of samples was analyzed by Fluoride Ion Selective Electrode. The mean ± SD fluoride content of the bottled waters was 0.202 ± 0.00152 mg/L with a range from 0.039 to 0.628 mg/L which was lower than the accepted limits for fluoride content of drinking water (1 mg/L). This finding suggested that in the region which water has high fluoride content, drinking bottled water is preferred to drinking tap water, as it could lower the risk of fluorosis. However, the risk of dental caries increases in people who mainly drink bottled waters; thus, they should use fluoride supplements.

## Introduction

Fluoride has a pivotal role in prevention of dental caries, and its deficiency may initiate some dental problems (1). Conversely, excessive exposure to fluoride may lead to a number of adverse effects, ranging from mild dental fluorosis (2) to crippling skeletal fluorosis (3). Although fluoride’s primary benefit is resulted by its topical contact with teeth, adequate intake of fluoride is essential for dental health, especially in children (4). Since water is the main source of ingested fluoride for human, knowledge of the fluoride levels in drinking water is an important issue (5). Keeping the concentration of fluoride in the range of 0.5 to 1.5 mg/L in water has been recommended by the World Health Organization (6); consequently, many countries set 1.5 mg/ L as the maximum contaminant level (MCL) for fluoride in drinking water (7). It has been demonstrated that the content of fluoride in drinking water in Iran is generally high, and in some regions (e.g. southern parts) this matter has leaded to dental fluorosis (8). However, dental carries due to fluoride deficiency is the other matter of concern in some other parts of Iran (9).

Traditionally, tap water has made an important contribution to total water intake, and 40%-50% of daily water intake originates from this source when the air temperature is less than 21°C (10).

However, more recently, there has been a trend to drink more natural beverages (11), a developing passion with fitness, greater travel and access to refreshments, as well as to move towards a greater consumption of food outside the home. These changes together with public concern about the taste and quality of water supplies and their potential contamination have resulted in many people’s turning to bottled water (12). According to a commercial report in 1995, 800 million liters of bottled water were consumed in the UK and this figure had increased to 1,390 million liters in 2000 (13).

The concentration of some elements such as calcium, sodium, iron, silver and aluminum, in bottled drinking waters is regulated in some countries (14); yet, there is no regulation regarding the fluoride content of bottled water in Iran. Manufacturers are encouraged to list the nutritional contents of their products, but stating the fluoride levels of bottled water on labels is not legally required. The fluoride content of bottled waters can be highly variable, and this may have oral health implications for those individuals, especially children, who drink bottled water as their primary source of drinking water (15). A first step toward evaluating how bottled water consumption might affect fluoride exposure is to determine the fluoride content of bottled waters.

An ion-selective electrode is the most cost-effective, efficient and reliable analytical method for determination of the fluoride level in various samples (16). By means of this method it is possible to measure the total amount of free and complex-bound fluoride dissolved in water. The method can be used for water containing at least 20 μg/L fluorides (17).

For an individual, fluoride exposure (mg/kg/day) via drinking-water is determined by the fluoride level in the water and the daily water consumption. Water consumption increases with temperature, humidity, exercise and state of health, and it is modified by other factors including diet (18). The objective of the present study was to provide up-to-date information on the fluoride content of a series of bottled drinking waters (including the types of spring, natural and mineral) currently in market in Iran. The obtained data would be of use to dentists both in clinical practice and public dental health, and it would also provide them with current information necessary for advising on fluoride supplementation to people. 

## Experimental

*Apparatus*

A Metrohm 692 pH/Ion Meter with a Metrohm fluoride ion selective electrode (6.0502.150) coupled with Metrohm reference electrode Ag/AgCl (6.0729.100) was used for fluoride measurements.

*Chemicals, reagents and materials*

CTDA (trans-1,2-diaminocyclohexane N,N,N΄,N΄-tetraacetic acid), NaCl, NaF, NaOH and glacial acetic acid were of analytical reagent (AR) grade and were purchased from Merck (Germany).

*Sampling*

Eighteen brands of bottled drinking waters were selected from those available in major supermarkets and grocery stores (May 2007, Tehran, Iran). Six bottles of each brand with different batch numbers and date of bottling were purchased. The bottles were made of polyethylene terphethalate (PET).

*Fluoride standards and analysis*

A series of eight fluoride standards ranging from 0.020 mg/L to 1.00 mg/L were prepared using sodium fluoride in deionized water. Total ionic strength adjusting buffer II (TISAB II) was prepared by mixing 4 g CDTA, 57 mL glacial acetic acid and 58 g NaCl in about 500 mL deionized water, adjusting the pH to 5-5.5 by adding 5 M NaOH (200 g/L) and then diluting to 1 liter with deionized water, according to application bulletin NO.82/3e (19). This range of fluoride ion concentrations ensured that the Ion Meter was properly calibrated for the quantitative determination of fluoride in water samples. The Metrohm Ion Meter was calibrated using the eight standards in the direct mode.

*Fluoride determination*

After shaking the bottle of water, a 10.0 mL sample solution was taken and mixed with 10.0 mL of TISAB II; after that, fluoride concentrations of all 108 samples were determined in duplicate using fluoride ion selective electrode. One batch number (out of six) for each of the 18 bottled drinking waters was randomly selected and the samples re-analyzed to assess the reliability of the method. 

*Data management*

Final calculation of the fluoride content of samples from the ion selective electrode (ISE) meter readings (in mV) was carried out in Microsoft Excel 2003 using logarithmic regression. SPSS (Statistical Package for Social Sciences, ver.10.1) was used to derive descriptive data. 

## Results and Discussion

**Figure 1:**
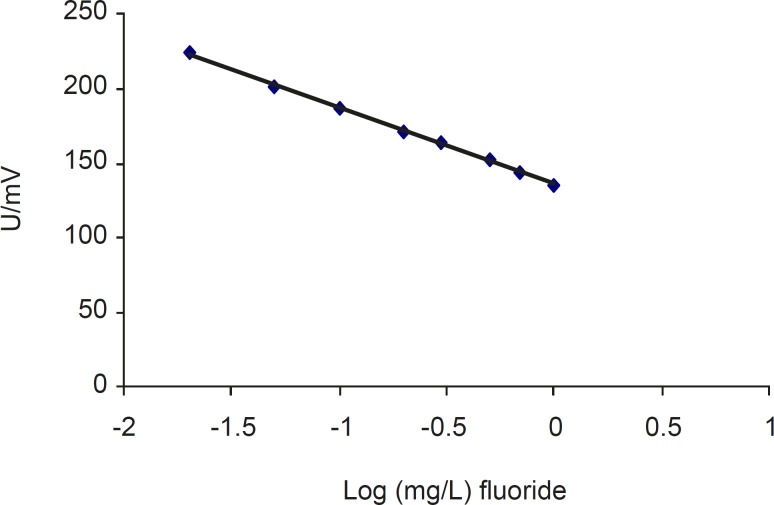
The calibration curve for determining fluoride in bottled drinking waters

The linear calibration curve was obtained by plotting mV vs. log mg/L fluoride content in the linear calibration curve was obtained by plotting mV vs. log mg/L fluoride content in bottled drinking waters (Figure 1). The fluoride calibration curve gave the following equation: 

*y *= -51.207*x *+ 86 135.91 (R^2^ = 0.9986) (Eq. 1)

Table 1 lists the most common brands of bottled drinking water according to fluoride concentration (mg/L) and the label-claimed fluoride content (if any). The mean ± SD fluoride content of the 18 bottled drinking water brands was 0.202 ± 0.00152 mg/L, ranging from 0.0396 to 0.628 mg/L. Parsi^®^ and Dimeh^®^ had the highest mean concentrations of 0.617 and 0.586 mg/L, respectively. The lowest concentration of fluoride was found in Anahita^®^ (0.040 mg/L). 

The quality of labeling varied so that 5 out of 18 bottled water samples did not claim the fluoride concentration on the label (Table 1); moreover, of the 13 labeled samples only one had a precise value for fluoride content. For two samples, i.e. Nava^®^ and Desani^®^ the claimed fluoride concentration was 3.5 and 5.5 folds, respectively as much as that measured in this study; for two other samples, i.e. Bisheh^®^ and Dimeh^®^ the claimed fluoride concentration on the label was 3.9 and 1.95 folds, respectively below those measured (Table 1). 

ANOVA model was applied to the further analysis to determine statistical significance between samples. Data showed a significant difference [F (17, 90) = 16655.628, P < 0.05] between all samples. According to this study, samples were placed in 12 groups based on their fluoride contents. 

**Figure 2:**
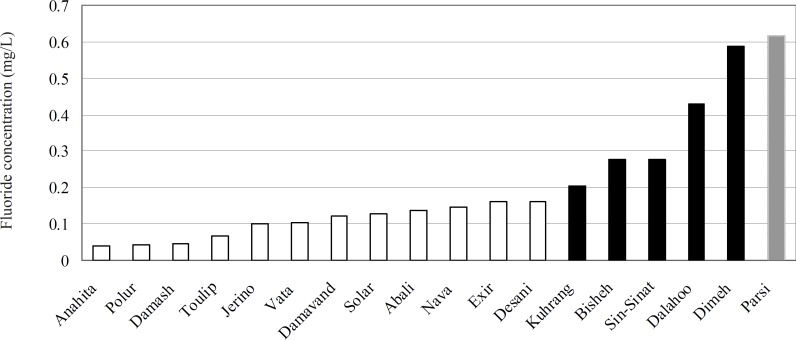
Fluoride content of bottled drinking water in Iran. White bar is related to bottled waters from the Alborz Mountains while black bar considered the one from the Zagros Mountains. Gray bar is related to bottled waters with unknown origin

As shown in Table 1, the fluoride contents of the studied samples showed a wide variation, ranging from 0.039 to 0.629 mg/L. The broad range of fluoride content in water samples is probably due to the contact of water with soils and rocks having a variety of fluoride contents. Figure 2 shows the fluoride content of commercially available bottled waters. There are two major mountain ranges in Iran each of them has its own character, namely Alborz which runs from west to east, and Zagros which extends from northwest to southeast. The results of this study revealed that bottled waters from Zagros Mountains have greater fluoride content than those from Alborz, assuming higher fluoride content of regions in Zagros through which mineral water flows compared to those in Alborz range. This result is in accordance with several previous studies about prevalence of fluorosis in the southern regions of Iran (8, 9, 20, 21). 

High levels of fluoride at concentrations up to 10 mg/L result in dental fluorosis (yellowish or brownish striations or mottling of the enamel), while low levels of fluoride, i.e. less than 0.1 mg/ L could increase the possibility of dental caries (22); however, poor nutritional status is also an important contributory factor to dental caries. The level of dental caries (measured as the mean number of decayed, missing or filled teeth) falls from seven at a fluoride concentration of 0.1 mg/L to around 3.5 at a fluoride concentration of 1.0 mg/L.

**Table 1:** The fluoride content of 18 bottled water

**No. **	**Bottled water brand **	**Fluoride concentration** ^*^ ** (Mean ± SD) (mg/L) **	**Range (mg/L) **	**Labeled claimed **fluoride (mg/L)	**Source of water **	**pH **
1	Anahita	0.040 ± 3.78 ×10^-4^	0.039 - 0.041	0.07	Haraz (Alborz)	7.2
2	Polur	0.044 ± 3.78 ×10^-4^	0.043 - 0.044	0.07	Polur (Alborz)	7.4
3	Damash	0.047 ± 8.23 ×10^-4^	0.046 - 0.048	< 0.2	Gilan (Alborz)	7.3
4	Toulip	0.066 ± 1.29 ×10^-3^	0.064 - 0.067	NL	Sari (Alborz)	7.5
5	Jerino	0.101 ± 1.78 ×10^-3^	0.099 - 0.102	NL	Polur (Alborz)	8
6	Vata	0.102 ± 2.42 ×10^-3^	0.099 - 0.106	0.11	Sabalan (Alborz)	7
7	Damavand	0.123 ± 7.53 ×10^-4^	0.122 - 0.124	0.2	Alborz (Alborz)	7.3
8	Solar	0.128 ± 5.16 ×10^-4^	0.128 - 0.129	0.37	Damavand (Alborz)	7.7
9	Abali	0.138 ± 2.16 ×10^-3^	0.135 - 0.141	NL	Haraz (Alborz)	NL
10	Nava	0.145 ± 1.16 ×10^-3^	0.144 - 0.146	0.5	Damavand (Alborz)	7.6
11	Exir	0.162 ± 2.63 ×10^-3^	0.159 - 0.166	NL	Tehran (Alborz)	7.8
12	Desani	0.162 ± 4.35 ×10^-3^	0.155 - 0.167	0.6 -1.1	Mashad (Alborz)	7.2
13	Kuhrang	0.204 ± 1.78 ×10^-3^	0.202 - 0.207	0.23	Sanandaj (Zagros)	7.5
14	Bisheh	0.276 ± 3.06 ×10^-3^	0.274 - 0.280	0.07	Lorestan (Zagros)	7.06
15	Sin-Sinat	0.276 ± 1.16 ×10^-3^	0.275 - 0.278	NL	Shahrekord (Zagros)	NL
16	Dalahoo	0.429 ± 2.28 ×10^-3^	0.426 - 0.432	0.5	Kermanshah (Zagros)	7.4
17	Dimeh	0.586 ± 2.09 ×10^-3^	0.583 - 0.588	0.3	Shahrekord (Zagros)	7.5
18	Parsi	0.617 ± 1.13 ×10^-2^	0.596 - 0.628	0.72	Unknown origin	7.9
-	Total	0.202 ± 1.70 ×10^-1^	0.039 - 0.628	-	-	7.46

NL: not labeled; * Values of 6 individual samples determination

Conversely, at a fluoride concentration of 1 mg/L about 20% of children would have evidence of dental fluorosis, but this fluorosis is of a mild degree of severity and would not be cosmetically obvious to the children or their parents (22). Moreover, it has been reported that a suitable substitution between caries and fluorosis appears to occur at around 0.7 mg/L fluoride (10). Unfortunately the result of this study showed that bottled drinking waters had the fluoride content below 0.7 mg/L; thus, it is important to be aware of fluoride intake especially in children who use bottled water as the main source of drinking water.

It has been demonstrated that fluoride concentration of drinking water in southern part of Iran is around 2 mg/L, which is higher than the permitted limit (0.6-1.2 mg/L), as a result the prevalence rate of dental fluorosis is high (8); therefore, high levels of fluoride should be prevented (9). Elimination of excessive fluoride from drinking water may be difficult and expensive; hence, bottled drinking water, which has lower-than-limit fluoride content based on our study, could be suggested as a main source of drinking water in these areas to optimize their fluoride intake for optimal oral health.

Additionally, this study showed that 72% of water samples had claimed the fluoride content on the labels. Eighty nine percent of labeled bottled drinking water samples contained fluoride under or over the labeled claim, and only 5% of them displayed precise values for fluoride content. This difference may be attributed to different sampling time and method of analysis; consequently, health professionals should be aware of the fact that labeled values may not be reliable.

The results of this investigation indicate that the bottled drinking waters in Iran are insufficient in fluoride content, compared to the level recommended by WHO (6); accordingly, dentists or other health care providers should be mindful that these persons need fluoride supplement; if these people mainly use bottled water, they might suffer from fluoride deficiency and dental caries (16).

It is also important that the consumers have accurate information on the fluoride content of the water that they drink. With this it may be advisable for bottled waters to be assayed at least twice per year for their fluoride content by an independent organization. There is no strict regulation on the labeling of fluoride contents of bottled drinking waters in Iran; thus, appropriate regulation seems to be necessary.

## References

[1] Griffin SO, Regnier E, Griffin PM, Huntley V (2007). Effectiveness of fluoride in preventing caries in adults. J. Dent. Res.

[2] Williams DM, Chestnutt IG, Bennett PD, Hood K, Lowe R (2006). Characteristics attributed to individuals with dental fluorosis. Comm. Dent. Health.

[3] World Health Organization IPCS (2002) Fluorides: International Programme on Chemical Safety (Environmental Health Criteria 227).

[4] Hardman MC, Davies GM, Duxbury JT, Davies RM (2007). A cluster randomized controlled trial to evaluate the effectiveness of fluoride varnish as a public health measure to reduce caries in children. Caries Res.

[5] Shailaja K, Johnson ME (2007). Fluorides in ground water and its impact on health. J. Environ. Biol.

[6] World Health Organization (1996). WHO Guidelines for Drinking-water Quality: Health Criteria and other Supporting Information.

[7] Hurtado R, Gardea-Torresdey J, Tiemann KJ (2000). Fluoride Occurrence in Tap Water at Los Altos de Jalisco in the Central Mexico Region. http://www.engg.ksu.edu/HSRC/00Proceed/gardea_torredey1.pdf.

[8] Ramezani GHH, Valaie NB, Eikani H (2004). Prevalence of DMFT and fluorosis in the students of Dayer city (Iran). J. Indian Soc. Pedod. Prev. Dent.

[9] Meyer-Lueckel H, Bitter K, Shirkhani B, Hopfenmuller W, Kielbassa AM (2007). Prevalence of caries and fluorosis in adolescents in Iran. Quintessence Int.

[10] Heller KE, Sohn W, Burt BA, Eklund SA (1999). Water consumption in the United States in 1994-96 and implications for water fluoridation policy. J. Public Health Dent.

[11] McGuire S (1989). Fluoride content of bottled water. New Eng. J. Med.

[12] Bedford AM (2002). Liquid refreshments: Beverages and health. Nut. Bulletin.

[13] Zohouri FV, Maguire A, Moynihan PJ (2003). Fluoride content of still bottled waters available in the North-East of England, UK. Br. Dent. J.

[14] Flaitz CM, Hill EM, Hicks MJ (1989). A survey of bottled water usage by pediatric dental patients: implications for dental health. Quintessence Int.

[15] Ahmed R, Hussain M, Tanwir R, Qureishi SA (2004). Monitoring of fluoride and iodide levels in drinking water using ion selective electrode. The Nucleus.

[16] Slooff W (1988). Basis Document Fluoriden.

[17] Slooff W, Eerens HC, Janus JA, Ros JPM, Janssen PJCM, Knaap AGAC, Lagas P, Matthijsen AJCM, Reijnders HFR, Struijs J, Wiel HJ van de, Anzion CJM, Eerden LJM van der, Duiser JA, Hollander JCT, de Jong P, van der Woerd KF (1988). Basis Document Fluoriden. Report No. 758474005.

[18] World Health Organization (1986). Appropriate Use of Fluorides for Human Health.

[19] Metrohm lnc Determination of Fluoride with the Ion-selective Electrode. Application Bulletin.

[20] Imandel K, Khodabandeh A, Mesghaly A, Firozian H (1997). Epidemiology of fluorosis in the Borazjan area of Iran I. Fluoride content in drinking water. Southeast Asian J. Trop. Med. Pub. Health.

[21] Meyer-Lueckel H, Paris S, Shirkhani B, Hopfenmuller W, Kielbassa AM (2006). Caries and fluorosis in 6- and 9-year-old children residing in three communities in Iran. Comm. Dent. Oral Epidem.

[22] Edmunds WM, Smedley PL (1996). Ground water geochemistry and health: an overview. Environmental geochemistry and health with special reference to developing countries.

